# Rivaroxaban Rebound Acute Coronary Event: A Post Marketing Experience

**DOI:** 10.4021/cr294w

**Published:** 2014-01-02

**Authors:** Ajay Agarwal, Amish Patel, Omar Mufti, Yaser Jbara, Ali Abdul Jabbar

**Affiliations:** a Cardiology Division, Department of Internal Medicine at Wright State University Boonshoft School of Medicine, Dayton, OH, USA; b Cardiology Department, Dayton VA Medical Center, Dayton, OH, USA

**Keywords:** Rivaroxaban, Anticoagulation, Acute coronary syndrome

## Abstract

We present a 65-year-old male who received rivaroxaban therapy prior to and after left knee replacement surgery. The patient developed generalized weakness soon after stopping rivaroxaban. An electrocardiogram showed acute infero-lateral ischemia and an echocardiogram reported an akinetic antero-apical wall segment, an apical clot and a reduced systolic function. A subsequent coronary angiogram revealed two-vessel coronary artery thrombosis. The case illustrates a temporal relationship of coronary thrombosis following rivaroxaban cessation.

## Introduction

This case discusses the relationship of rivaroxaban cessation and adverse events. The patient underwent a left knee replacement surgery; rivaroxaban was used prior to surgery and discontinued 12 days post-operatively. A pre-operative myocardial stress test was normal. After rivaroxaban cessation, the patient developed an acute coronary event.

## Case Report

A 65-year-old male with hypertension and hyperlipidemia complained of generalized weakness after his left knee replacement 2 weeks ago. Physical exam was unremarkable except a borderline-low blood pressure.

**Figure 1:**
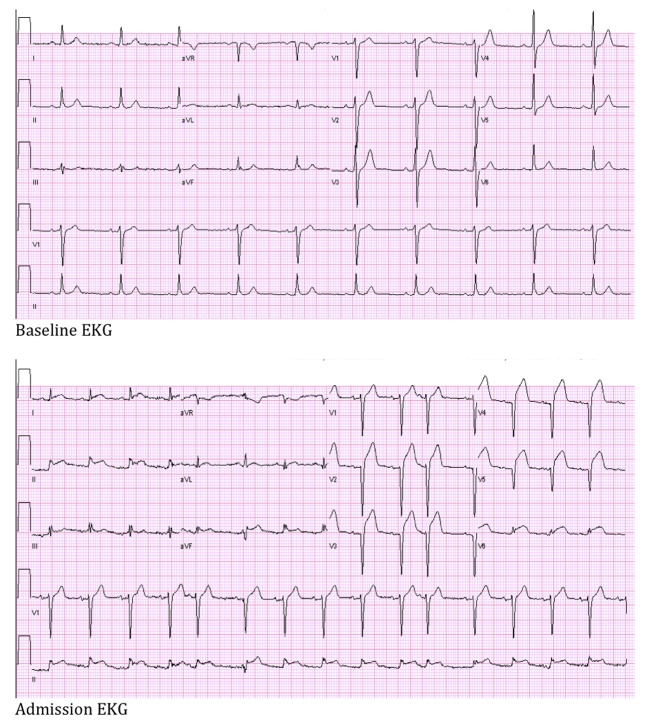
Baseline and admission electrocardiogram.

His electrocardiogram showed sinus rhythm, ST segment elevation with deep Q-waves in antero-septal precordial leads and isolated ST segment elevation in infero-lateral leads. The patient used rivaroxaban (Xeralto)™ prior to his surgery that was stopped 12 days post-operatively. He was referred immediately for cardiac catheterization, and his blood pressure responded well to saline bolus (Fig. 1).

A limited echocardiogram showed akinetic antero-apical segment (left anterior descending artery (LAD) territory) with apical clot and severely reduced left ventricular (LV) systolic function suggesting a recent anterior wall myocardial ischemia.

A pre-operative myocardial perfusion-scan prior to his knee surgery showed a satisfactory LV systolic function and no evidence of reversible myocardial ischemia.

**Figure 2:**
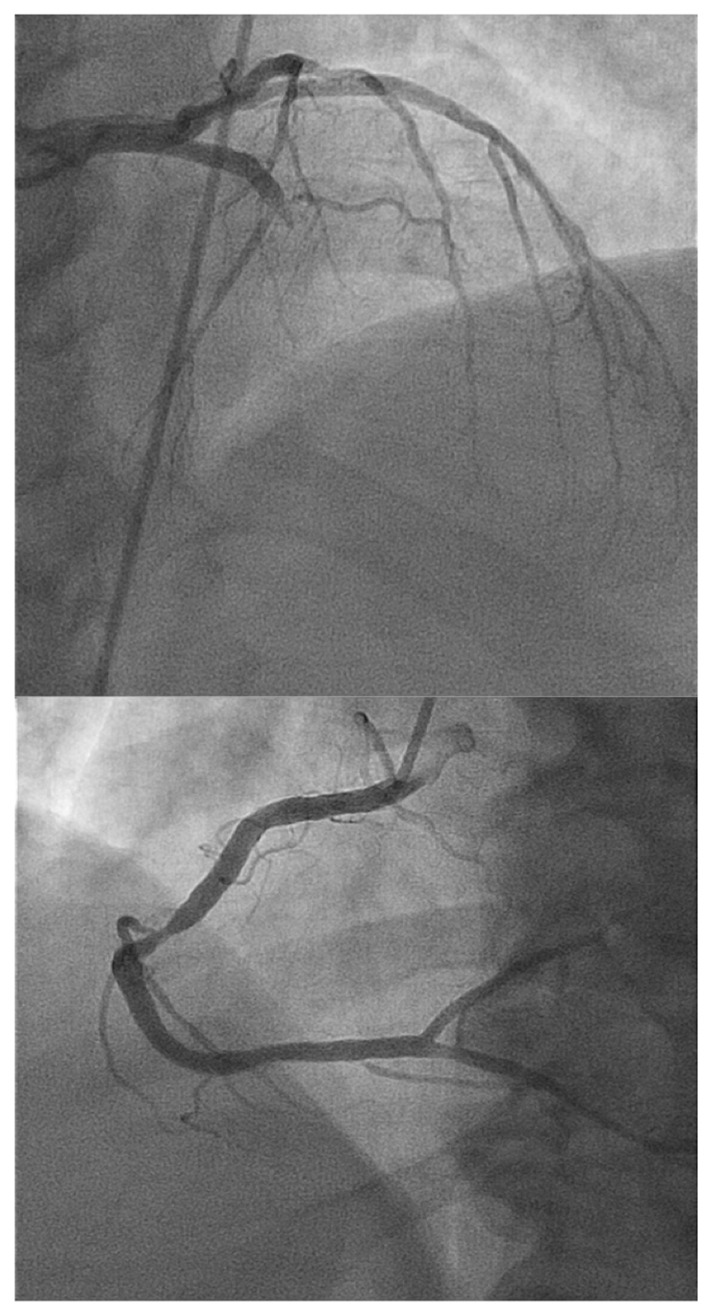
Total occlusion of mid LAD and culprit lesion in mid right coronary artery.

Coronary angiograms revealed a double vessel coronary disease with 80% lesion in mid right coronary artery and 100% total occlusion of mid LAD. No left-to-left or right-to-left collaterals to LAD were seen. LV angiogram was not performed due to the presence of apical clot on echocardiography (Fig. 2).

In view of his late presentation, the LAD lesion was not considered acute but recent thus deemed to have no benefit of primary percutaneous coronary intervention, and percutaneous coronary intervention of right coronary artery was preceded. The intervention was successful with TIMI 3 flow post stenting. The patient tolerated the procedure well. Day one post-operatively, the patient was asymptomatic with stable vital signs. Shortly after, he witnessed a sudden cardiac arrest of pulseless electrical activity followed by ventricular fibrillation not responding to conventional treatment. A bedside echocardiogram revealed a large pericardial effusion and non-contractile ventricles suggestive of LV wall rapture. The patient was pronounced dead the same day after unsuccessful resuscitation.

## Discussion

The novel anticoagulants are considered advancement in medical therapy where oral drugs are used conveniently without frequent monitoring. Being at the convergence point of the intrinsic and extrinsic clotting pathways, factor Xa has been the target for these novel anticoagulants therapies [1].

Binding to the negatively charged phospholipid surfaces of the activated platelets, together with factor Va and factor Xa, leads to the formation of the prothrombinase complex, the central prothrombin activator, which finally converts prothrombin to thrombin, the major player in the clotting process. It has a rapid onset and a reversible action on the inhibition of factor Xa activity as it binds directly to the active site of factor Xa via the S1 and S4 pockets [2].

Although the FDA has approved the use of rivaroxaban in non-valvular Afib and venous thrombo-embolism secondary prevention, a black box warning has been issued about the increased thrombotic events with cessation of therapy.

In phase III clinical trial for rivaroxiban use for thromboprophylaxis after hip arthroplasty, cardiovascular events were similar between the rivaroxaban group and enoxaparin group during the study. However, during follow-up, eight events (four coronary events) occurred in seven patients in the rivaroxaban group compared to one event in the enoxaparin group [3].

In ROCKET AF trial, the increased risk of stroke and non-CNS embolism observed in rivaroxaban group compared with warfarin group after the end of the study was explained by the absence of therapeutic anticoagulation coverage during such a transition and likely represents the intrinsic stroke rate of patients at moderate to high risk without therapeutic anticoagulation. Invasive procedures were the most common reasons for anticoagulant therapy interruptions with a median duration of 6 days [4].

Is rivaroxaban discontinuation associated with a pro-thrombotic rebound effect? It seems counterintuitive to explain the excess strokes in rivaroxaban patients as a result of under anticoagulation in the post-trial period and the high bleeding risk in these patients at the same time [5].

EINSTEIN investigators showed similar vascular events rivaroxaban compared to the standard therapy among the patients treated for venous thrombosis and pulmonary embolism [6, 7]. While the analysis of the RECORD trials 1, 2 and 4 suggests that rivaroxaban use may be associated with an increased risk of ischemic and cardiovascular events upon discontinuation of the drug [8].

The rebound phenomenon has been hypothesized for warfarin, but in case of rivaroxaban, it is not clear yet. Two cases of coronary thrombosis have been reported within less than a week of traditional anti-coagulation discontinuation [9]. In other reports, 20 patients had acute myocardial infarction within 4 weeks after the discontinuation of coumadin. Only two had a prior history of myocardial infarction. The interval from the last dose of the drug to the recurrence of infarction averaged around 11 days with a total of seven fatalities with the recurrent attack [10].

These reports and others raised the concern about the presence of a hypercoagulable state of the blood incident to the release of anticoagulation in patients whose clotting has been significantly depressed by anti-coagulant drugs. Others suggested that the cause is a “catching up” of the obliterating coronary artery disease [11].

In our report, the non-invasive work-up did not reveal any evidence of a significant coronary artery disease but within few days of rivaroxaban cessation, our patient presented with a sub-acute coronary thrombosis that ended up in a devastating event.

The FDA analytic review of the death events, and specifically myocardial infarction as a cause of mortality, showed no difference between rivaroxaban and warfarin. Those events were numerically in favor of rivaroxaban [12].

Van Thiel et al raised the concern about this cardiovascular rebound phenomenon recommending additional clinical validation to establish the safety of rivaroxaban [8].

Although it seems to be wise to minimize the interruption in anticoagulant therapy, the clinical benefit from bridging anticoagulation in patients who are transitioned from rivaroxaban to warfarin is still unclear. Bridging with ASA is another consideration.

In conclusion, acute coronary event is a possible post marketing adverse event after discontinuation of rivaroxaban therapy. Individual risk factors would increase the risk of such adverse events. Alternative therapy is yet to be considered in these high-risk patients.
